# Gigantic electric-field-induced second harmonic generation from an organic conjugated polymer enhanced by a band-edge effect

**DOI:** 10.1038/s41377-019-0128-z

**Published:** 2019-01-30

**Authors:** Shumei Chen, King Fai Li, Guixin Li, Kok Wai Cheah, Shuang Zhang

**Affiliations:** 1 School of Physics and Astronomy, University of Birmingham, Birmingham, B15 2TT UK; 2 Department of Materials Science and Engineering, Shenzhen Institute for Quantum Science and Engineering, Southern University of Science and Technology, Shenzhen, 518055 China; 3 Department of Physics, Hong Kong Baptist University, Kowloon Tong, Hong Kong China

**Keywords:** Polymers, Nonlinear optics

## Abstract

Electric-field-induced second harmonic generation (EFISH), a third-order nonlinear process, arises from the interaction between the electric field of an external bias and that of two incident photons. EFISH can be used to dynamically control the nonlinear optical response of materials and is therefore promising for active nonlinear devices. However, it has been challenging to achieve a strong modulation with EFISH in conventional nonlinear materials. Here, we report a large tunability of an EFISH signal from a subwavelength-thick polymer film sandwiched between a transparent electrode and a metallic mirror. By exploiting the band-edge-enhanced third-order nonlinear susceptibility of the organic conjugated polymer, we successfully demonstrate a gigantic EFISH effect with a modulation ratio up to 422% V^−^^1^ at a pumping wavelength of 840 nm. The band-edge-enhanced EFISH opens new avenues for modulating the intensity of SHG signals and for controlling nonlinear electro-optic interactions in nanophotonic devices.

## Device offers significant laser beam control

The properties of a laser beam can be strongly controlled by varying the voltage applied to a device made of an organic polymer sandwiched between electrodes. Nonlinear materials are often used to change laser beam properties, such as its frequency. But controlling this change has been challenging when using conventional nonlinear materials, like optical crystals. Shuang Zhang of the University of Birmingham, UK, Guixin Li from Southern University of Science and Technology, China and colleagues fabricated a device made of a 100 nanometre(nm)-thin film of the organic polymer polydioctylfluorene sandwiched between a 50 nm-thick layer of indium tin oxide and a 100 nm-thick layer of aluminum. Varying the voltage applied to the outer electrodes controls the frequency of the outgoing laser beam at second harmonic frequency, when the incoming beam is made to fall on the device at a 45° angle. The study’s findings could have implications for the future development of electro-optic modulators.

## Introduction

In nonlinear optics, it is well-known that the frequency conversion processes depend on both the chemical composition of the material and the spatial symmetry of the optical crystal^1,2^. Symmetry is especially important for second-order nonlinear processes. For example, the second order susceptibility *χ*^(2)^ is forbidden in centro-symmetric materials in the electric dipole approximation. Therefore, second harmonic generation (SHG) has been widely explored in natural crystals with broken inversion symmetry. Although the inversion symmetry of conventional optical crystals can be broken by introducing a stressor layer^3^, the modulation depth of the nonlinear optical susceptibility of the hybrid system is very limited. With artificial photonic structures, such as liquid crystals, photonic crystals, metamaterials and metasurfaces^4–6^, both local and global symmetries can be readily engineered to boost the SHG efficiency through quasi-phase matching^7–10^, plasmonic and magnetic resonances^11–15^, and continuous control of nonlinearity phase^16^.

The dynamic control of nonlinear optical signals may have important applications in optical modulation and switching. Various switching technologies based on Kerr or free-carrier nonlinearities in semiconductor materials have been developed using all-optical control schemes. Alternatively, the control of nonlinear optical signals can be realized using electro-optic interactions. For example, electric-field-induced SHG (EFISH), which was proposed in the early 1960s, provides an alternative route for designing nonlinear optical modulators^17^. In the EFISH process, an external static electric field can be mixed with the fundamental wave (FW) to produce SHG in nonlinear optical materials with large third-order susceptibilities. Because the symmetry of nonlinear optical materials has fewer restrictions in third-order processes compared to second-order processes, the EFISH process has been extensively exploited in various media, such as optical crystals^17,18^, electrolytic solutions^19^, metal–oxide–semiconductors^20^, organic devices^21^, silicon waveguides^22^, and photonic metamaterials^23–25^. It was demonstrated that the efficiency of the EFISH signal could be greatly improved by utilizing the backward phase matching technique in plasmonic metamaterials^25^ and quasi-phase matching in silicon waveguides ^22^. EFISH was recently realized by electrostatic doping in two-dimensional (2D) materials. Specifically, electrically tunable SHG was theoretically studied using plasmonic resonances in doped graphene nanoislands^26^ and experimentally realized based on strong exciton charging effects in monolayers of WSe_2_^27^. Electric-field-controlled SHG in 2D materials also has many limitations. For example, the SHG predicted in graphene nanoislands strongly relies on heavy doping of charge carriers and thus imposes critical requirements on the fabrication of graphene monolayers. In addition, the electric-field-enabled modulation depth of SHG from a WSe_2_ transistor was less than 3% V^−^^1^, which limits its potential applications as a nonlinear optical modulator.

Here, we demonstrate band-edge-enhanced EFISH from a subwavelength-thick organic conjugated polymer PFO (poly(9,9-di-n-dodecylfluorenyl-2,7-diyl) film sandwiched between two conducting layers—indium–tin–oxide (ITO) and aluminum, which serve as electrodes. The PFO polymer is a p-type π-conjugated polymer, which is usually used as a blue-emitting material with a bandgap energy of ~2.95 eV. It was reported that the PFO thin film has a high third-order susceptibility *χ*^(3)^ for the FW at near infrared wavelengths^28^. However, less attention has been paid to its nonlinear properties when the THG or SHG frequencies are close to the bandgap energy. In this work, we observe a large EFISH enhancement in PFO at a very low biased voltage when the energy of the FW is close to half of the peak absorption energy (3.18 eV) of PFO. The observed prominent EFISH effect in subwavelength-thick polymer devices with a modulation depth 422% V^−1^ may open new avenues for designing novel electro-optic modulators.

## Results

**Fig. 1:**
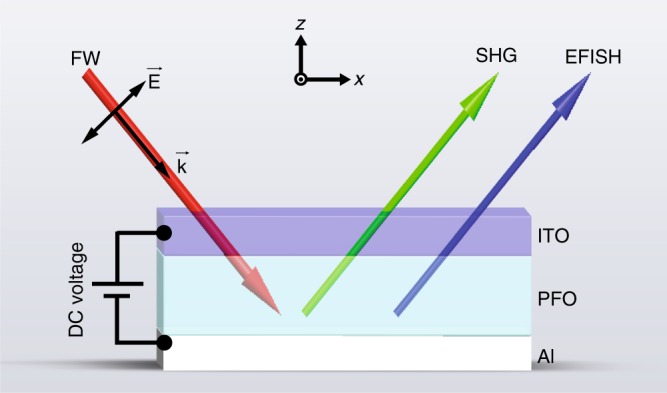
Schematic of electric-field-induced SHG (EFISH) from the ITO/PFO/aluminum device. For a fundamental wave (FW) with TM-polarization, obliquely incident onto the EFISH device, the intensity of the SHG waves can be modulated by applying a DC electric field. Under oblique incidence of the FW with TM-polarization (electric field parallel to *x*–*z* plane), EFISH comes from the coupling between the electric field of the incident light and that of an applied voltage using the third-order susceptibility of PFO. The electric field of the TE (electric field of light along *y*-axis)-polarized FW is perpendicular to that of the applied voltage, so EFISH is also forbidden

The EFISH device consists of a 100-nm-thick PFO thin film sandwiched between aluminum (Al, 100 nm) and ITO (50 nm) electrodes, as shown in Fig. 1. For PFO thicknesses less than 100 nm, there exists a high risk of a short circuit due to the roughness of the ITO layer. Thus, in this work, the PFO thickness deviates from the optimized value of 50 nm (see Supplementary materials for more discussions). Under normal incidence of the FW, SHG from amorphous PFO film is forbidden, as the second order susceptibility *χ*^(2)^ is negligible in the homogeneous film. However, SHG can be generated in homogenous PFO film under the condition of broken inversion symmetry from an oblique incidence of the FW. The reflected SHG intensity from the ITO/PFO/Al sandwiched photonic device can be described by:

1 $$\begin{array}{l}I_{2\omega } \propto \left[ {(\chi ^{(3)}E_{DC}{\mathrm{ + }}\chi ^{({\mathrm{2}})})E_\omega ^{\mathrm{2}}} \right]^{\mathrm{2}}\\ {\mathrm{ = }}((\chi ^{(3)}E_{DC})^2 + 2\chi ^{(2)}\chi ^{(3)}E_{DC})E_\omega ^4 + (\chi ^{(2)})^2E_\omega ^4\end{array}$$

where *E*_*ω*_ is the electric field of the FW; *E*_*DC*_ is the external electric field applied to the PFO thin film through the ITO and aluminum electrodes; and *χ*^(2)^ and *χ*^(3)^ are the effective second- and third-order susceptibilities of the PFO layer, respectively. *χ*^(2)^ arises from the generation of SHG at the Al–PFO or the ITO–PFO interfaces. In the second line of Eq. (1), the first and third terms describe the EFISH and the common SHG process, respectively, while the second term represents the interference between the two, which relies on both the *χ*^(2)^ and *χ*^(3)^ coefficients of the system. This is in contrast to most of the previous works on active materials where *χ*^(3)^ plays a dominant role^17–25^. Due to the coupling between the electric field of the FW and the DC field in the second term of Eq. (1), the intensity of EFISH can also be tuned by switching the sign of the applied DC electric field.

**Fig. 2:**
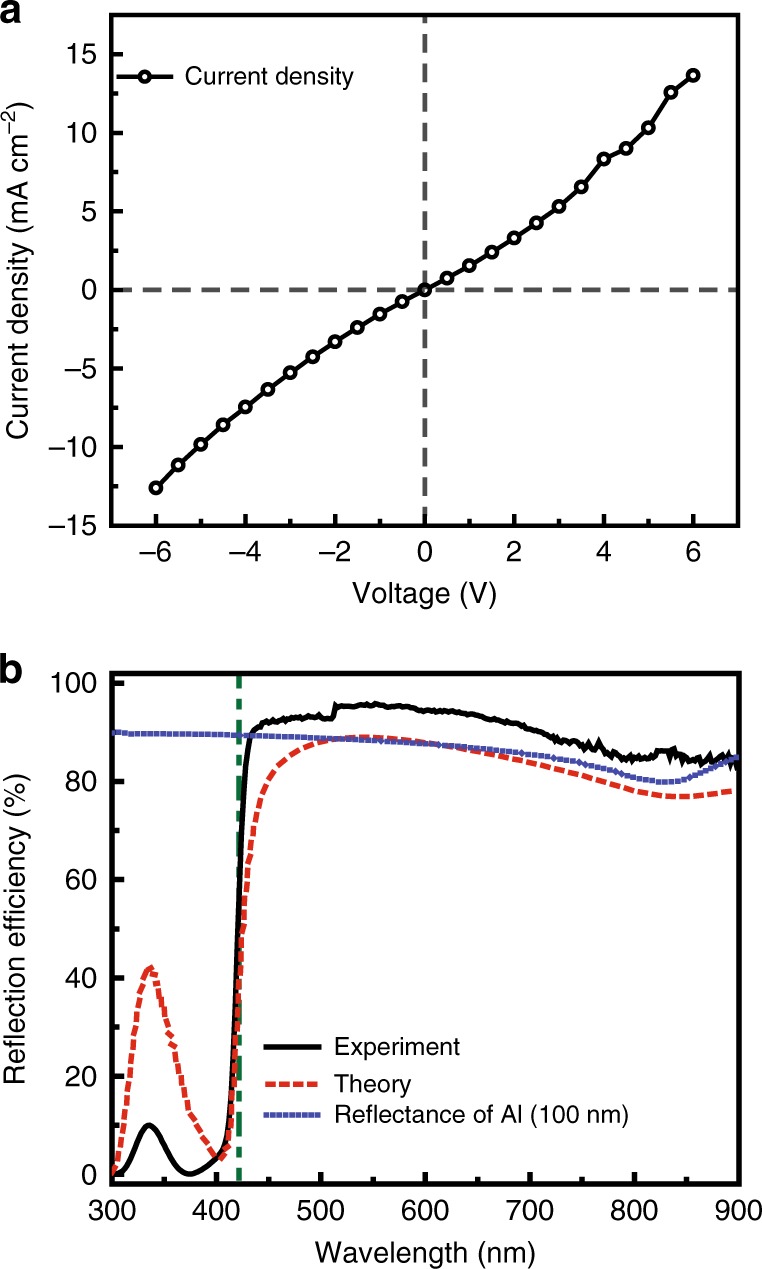
Electrical and optical properties of the ITO/PFO/aluminum device. **a** Current density as a function of applied voltage. ITO acts as either an anode or a cathode for positive and negative values of the applied voltage. **b** Measured reflection spectra of the EFISH device. The reflection efficiency drops quickly when the wavelength of the incident light is less than 430 nm due to the absorption of PFO. The dashed-dotted line at a wavelength of 420 nm corresponds to the peak position of the SHG in the nonlinear optical measurements. The red dashed line and the blue dotted line are the calculated reflectances of the EFISH device and a single aluminum layer with a thickness of 100 nm

To experimentally explore the modulation of the EFISH signal from the PFO thin film, we fabricated a 100-nm-thick homogeneous PFO thin film on top of ITO-coated glass using the spin-coating method, followed by thermal evaporation of a 100-nm-thick aluminum electrode. The triple-layer EFISH device is encapsulated in a nitrogen environment to avoid degradation of the PFO thin film. Both the electrical and linear optical properties of the EFISH device are characterized. The electrical properties are shown in Fig. 2a, where the current density (*I*) of the device is plotted as a function of the applied voltage (*V*). The positive/negative voltages can be applied by connecting the ITO electrode to the anode/cathode of a DC power supply. The current–voltage (*I*–*V*) curve is slightly asymmetric when the applied voltage is switched from positive to negative values, and vice versa, which is attributed to the common diode effect of the ITO/PFO/aluminum configuration. The reflection spectrum of the EFISH device is measured at an incident angle of 45° using a transverse magnetic (TM)-polarized FW. As shown in Fig. 2b, due to the strong absorption of the PFO thin film at wavelengths below 400 nm, the reflection efficiency of the device drops to less than 10%. The spectrum is featureless with a reflectivity above 80% for wavelengths between 400 and 900 nm, owing to the high reflectivity of the 100 nm aluminum film. The calculated reflectance of both the triple-layer device and the 100 nm aluminum film are obtained using the transfer matrix method^29^ with the measured refractive indices of PFO, ITO, and aluminum. The calculated results agree well with the measured results.

**Fig. 3:**
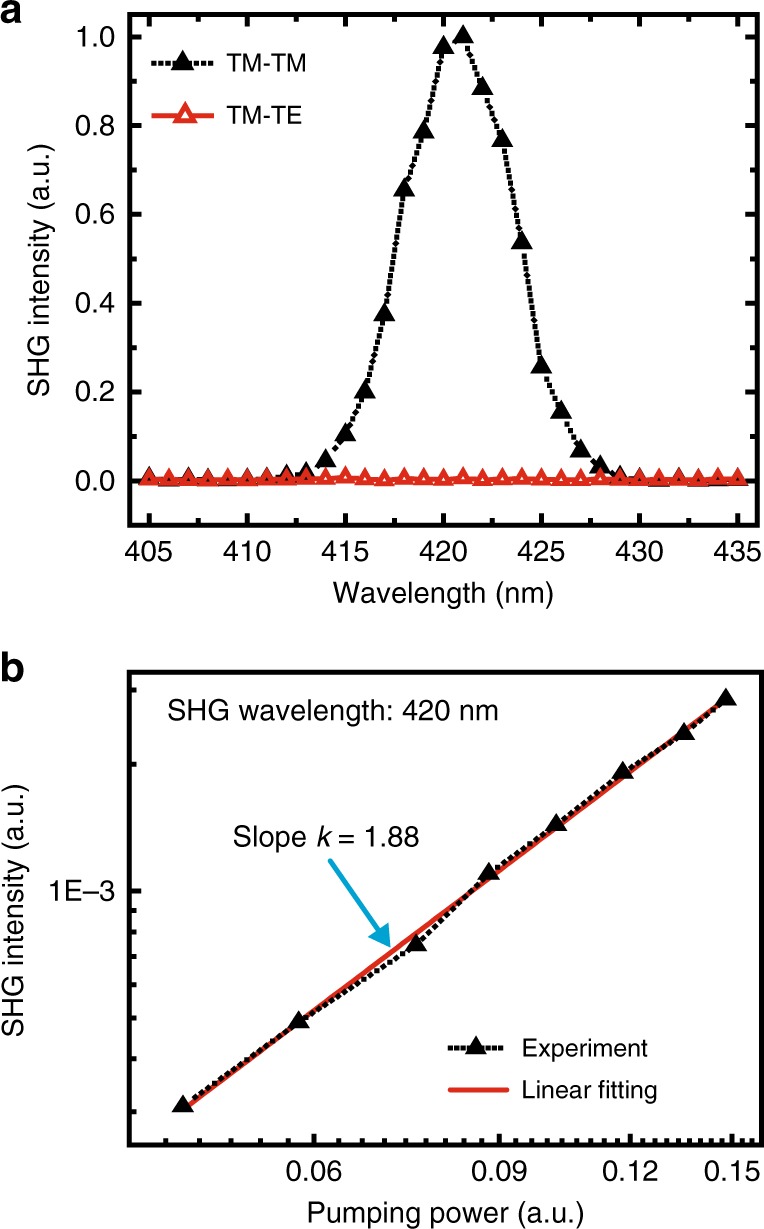
Nonlinear optical properties of ITO/PFO/aluminum without an applied voltage. The FW is obliquely incident onto the ITO/PFO/Aluminum system at angle of 45°. The thicknesses of ITO/PFO/Aluminum are 50/100/100 nm, respectively. The FW has TM polarization with an electric field parallel to the incident plane (*x*–*z*). **a** Polarization characteristics of the SHG at a wavelength of 420 nm, and the SHG spectra with the same polarization (TM) and cross-polarization states (TE, electric field along the *y*-axis) compared to that of the FW. It is found that the H-polarized SHG signal is much stronger than that with TE polarizations. **b** The SHG intensity as a relationship of the pumping power; the slope value of 1.88 indicates a second-order nonlinear optical process

**Fig. 4:**
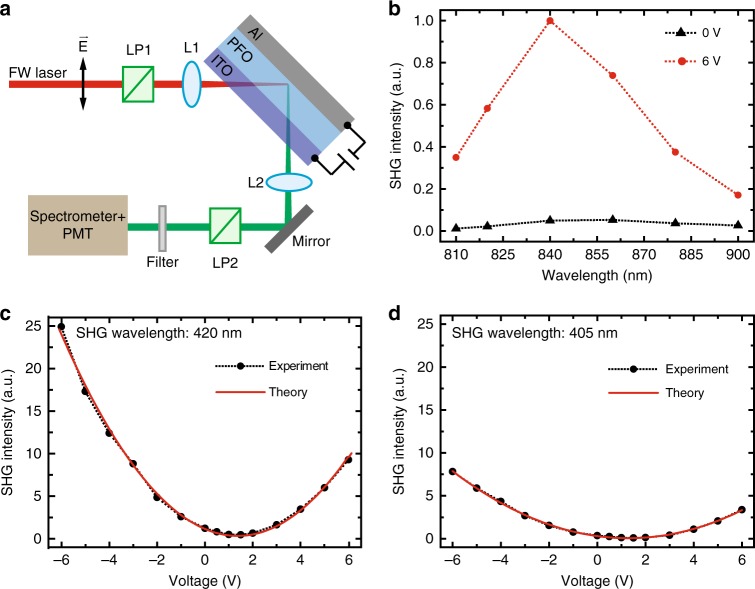
Nonlinear optical properties of ITO/PFO/aluminum with applied voltages. **a** Configuration of the SHG measurement. The TM-polarized FW is obliquely incident onto the ITO/PFO/aluminum device at an angle of 45°. L1 and L2 are lenses; LP1 and LP2 are polarizers. **b** Characterization of the spectral response of H-polarized SHG from ITO/PFO/aluminum with and without applied voltages. **c** The SHG intensity as a function of the applied voltages is plotted for the SHG wavelength at 420 nm. **d** The SHG intensity as a function of the applied voltages is plotted for the SHG wavelength at 405 nm. In the case of positive and negative voltages, the ITO layer serves as the anode and cathode, respectively. The fitting equations based on Eq. ( 1) are *y* = 0.4348*x*^2^ − 1.1944*x* + 1.1512 for SHG at 420 nm and *y* = 0.1470*x*^2^ − 3.7867*x* + 3.0622 for SHG at 405 nm. The retrieved relative values of the effective *χ*^(2)^ and *χ*^(3)^ of the EFISH device calculated from the fitting equations are 1.0729 and 0.6594 for SHG at 420 nm and 0.5543 and 0.3835 for SHG at 405 nm, respectively

We firstly studied the polarization states of the SHG signal generated from the EFISH device. For a TM-polarized FW at a wavelength of 840 nm, the SHG signal with the same polarization is much stronger than that of TE polarization (Fig. 3a), with a polarization ratio up to ~158. In addition, the power-dependent SHG intensity at a wavelength of 420 nm has a slope value of 1.88 (Fig. 3b), which is close to the theoretical value of 2.0, indicating a second-order nonlinear optical porcess. Next, SHG from the ITO/PFO/aluminum is characterized using a femtosecond laser with tunable wavelength output. The TM-polarized FW is obliquely incident onto the device after passing through a lens with a focal length of 150 mm. The central wavelength of 840 nm of the FW has a bandwidth of approximately 15 nm. SHG signals from the EFISH device without an applied DC voltage are first measured using an Andor spectrometer with a photomultiplier tube detector, as shown in Fig. 4a. For the FW at wavelengths from 810 to 900 nm, the wavelength-dependent intensity of the SHG, which should be proportional to the square of the modulus of the effective *χ*^(2)^, is experimentally characterized. Fig. 4b shows that the SHG efficiency exhibits a sharp peak at the fundamental wavelength of 840 nm. The resonant behavior of the SHG intensity can be attributed to the enhancement of in the effective *χ*^(2)^ when twice the energy of the FW is close to the absorption band of PFO^28^.

We next study electric-field-induced SHG from the ITO/PFO/aluminum device by applying a DC voltage (*U*) to the ITO and aluminum electrodes. As shown in Fig. 4a, the ITO layer can be used as either an anode (*U* > 0) or a cathode (*U* < 0). To avoid the damage of the EFISH device, the spectral measurement of the EFISH signal for a DC voltage only up to *U* = 6 V is carried out (red dot line with circles in Fig. 4b) for a TM-polarized FW at an incident angle of 45°. Compared to the case of *U* = 0 (black dot line with triangles in Fig. 4b), one can see that the applied electric field can greatly boost the SHG efficiency. To better understand the mechanism and the efficiency of the EFISH process, we plot the electric-field-induced SHG intensity versus the applied voltage *U* at the fundamental wavelengths of 840 and 810 nm in Fig. 4c, d, respectively. At the resonant wavelength of 840 nm (Fig. 4c), the SHG always has the highest efficiency for the EFISH device regardless of the applied DC electric field. When *U* is swept from 0 to 6 V, the SHG intensity initially drops to a minimum value of *I*_2*ω*_ = 0.489 (a.u.) at *U* = 1.5 V and then grows quickly to *I*_2*ω*_ = 9.29 (a.u.) at *U* = 6 V. This corresponds to an SHG modulation depth of *I*_2*ω*_ (*U* = 6)/[∆*U*·*I*_2*ω*_ (*U* = 1.5)]~422% V^−^^1^, with ∆*U* = 4.5 V in this case. This modulation depth is much higher than that in conventional EFISH devices or the electric-field-controlled SHG from WSe_2_^26^. Additionally, the modulation depth of the electric-field-controlled SHG from plasmonic metamaterials^23,24^ and WSe_2_^27^ is less than 0.9% V^−1^ and 3% V^−1^, respectively. If a reversed voltage is applied to the device, EFISH shows very different behavior; the SHG intensity continues to increase when *U* is swept from 0 to −6 V, and the measured modulation depth of the SHG has a negative value of ~−335% V^−1^. In comparison, the electric-field-controlled modulation depth of SHG in WSe_2_ devices is negative for both positive and negative biases. A very similar optical response was observed from the EFISH measurement at a nonresonant wavelength of 810 nm (Fig. 4d). The measured EFISH dependence over the applied electric field can be perfectly explained by Eq. (1), and the relative values of the effective *χ*^(2)^ and *χ*^(3)^ of the EFISH device can be retrieved by fitting the measured EFISH curves using Eq. (1), as shown in Fig. 4c, d (see Supplementary materials for more details). The ratio between the effective *χ*^(2)^ and *χ*^(3)^ is found to be 1.6271 and 1.4451 V m^−^^1^ for FWs at 840 and 810 nm, respectively.

## Discussion

We have shown that the EFISH device based on an organic conjugated polymer provides an excellent platform for electrically controlled SHG. The SHG efficiency with a frequency at the edge of the absorption band of PFO is greatly boosted due to the resonant nature of the *χ*^(3)^ of the organic conjugated polymer. To the best of our knowledge, the EFISH modulation depth of ~422% V^−^^1^ is the highest value ever reported. The maximum EFISHG efficiency of 1.92 × 10^−^^5^ was obtained at a wavelength of 420 nm when a 6.0 V external voltage was applied. In addition, the effective *χ*^(2)^ of the EFISH device and the *χ*^(3)^ of PFO were successfully retrieved, which sheds light on the mechanism of EFISH in the ITO/PFO/aluminum device. This work opens new avenues for designing electric field-controlled SHG based on organic conjugated polymers with a large modulation depth. It is expected that through the integration of plasmonic metamaterials and metasurfaces into the current EFISH device, the performance such as the modulation depth and efficiency of the SHG will be further enhanced, indicating great potential in applications such as electro-optic modulators.

## Materials and methods

### Sample fabrication

The blue-emission polymer PFO was used as the active material in this work. The PFO materials were purchased from Sigma-Aldrich. The molecular weights of PFO are Mw ≦ 20,000. PFO was dissolved in toluene solution at a concentration of 16 mg mL^−^^1^. PFO films with a thickness of 100 were fabricated by spin-coating onto a 0.7-mm-thick ITO-coated glass substrate.

### SHG measurement

SHG was measured using femtosecond (fs) laser (repetition frequency: 1 kHz, pulse duration: ~ 100 fs) output from an optical parametric oscillator at wavelengths from 810 to 900 nm. The power of the FW in Fig. 2, measured by a photodiode (Newport 818) connected to a lock-in amplifier, is a relative value. The averaged power of the FW in Fig. 4c, d is 10 mW. Laser light with a spot size of ~100 µm in diameter is obliquely incident onto the EFISH device after passing through a lens (N.A. = 0.05). The SHG signals from the EFISH device are collected by the second lens (N.A. = 0.05). After filtering the pumping laser, SHG is measured by a PI-Acton spectrometer with a photomultiplier tube detector.

## Supplementary information

Supplementary Materials for EFISH
