# Supplementary material for: Gigantic electric-field-induced second harmonic generation from an organic conjugated polymer enhanced by a band-edge effect
Source: Light Sci Appl. 2019 Jan 30;8:17. doi: 10.1038/s41377-019-0128-z (PMC6351641; doi:10.1038/s41377-019-0128-z)
Supplement: Supplementary file 1 — Supplementary Materials for EFISH [file 41377_2019_128_MOESM1_ESM.docx]

**Gigantic Electric-field-induced Second Harmonic Generation from an Organic Conjugated Polymer Enhanced by Band-edge Effect**

Shumei Chen^1†^, King Fai Li^2†^, Guixin Li^2*^, Kok Wai Cheah^3^, and Shuang Zhang^1*^

^1^School of Physics & Astronomy, University of Birmingham, Birmingham, B15 2TT, UK

^2^Department of Materials Science and Engineering, Shenzhen Institute for Quantum Science and Engineering, Southern University of Science and Technology, Shenzhen, 518055, China

^3^Department of Physics, Hong Kong Baptist University, Kowloon Tong, Hong Kong

*Email: ligx@sustc.edu.cn; s.zhang@bham.ac.uk

**Origin of EFISH**

**
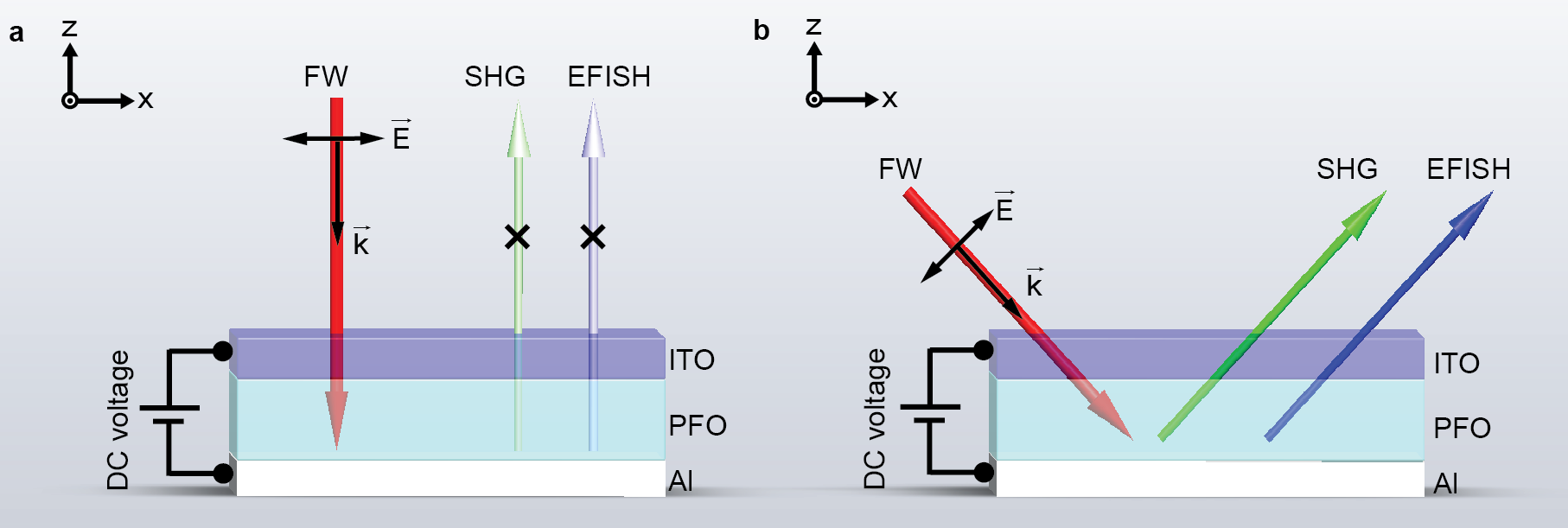
**

**Figure S1**. Schematic of electric field induced SHG (EFISH) from ITO/PFO/Aluminium device. For a fundamental wave (FW) with TM-polarization, obliquely incident onto the EFISH device, intensity of SHG waves can modulated by applying a DC electric field. (a) For normal incidence of FW, SHG and EFISH are negligible; (b) Under oblique incidence of FW with TM-polarization (electric field parallel to x-z plane), EFISH comes from the coupling between the electric field of incident light and that of an applied voltage using third-order susceptibility of PFO. The electric field of TE (electric field of light along y-axis)-polarized FW is perpendicular to that of the applied voltage, so EFISH is also forbidden.

**Nonlinear Optical Calculations**

**
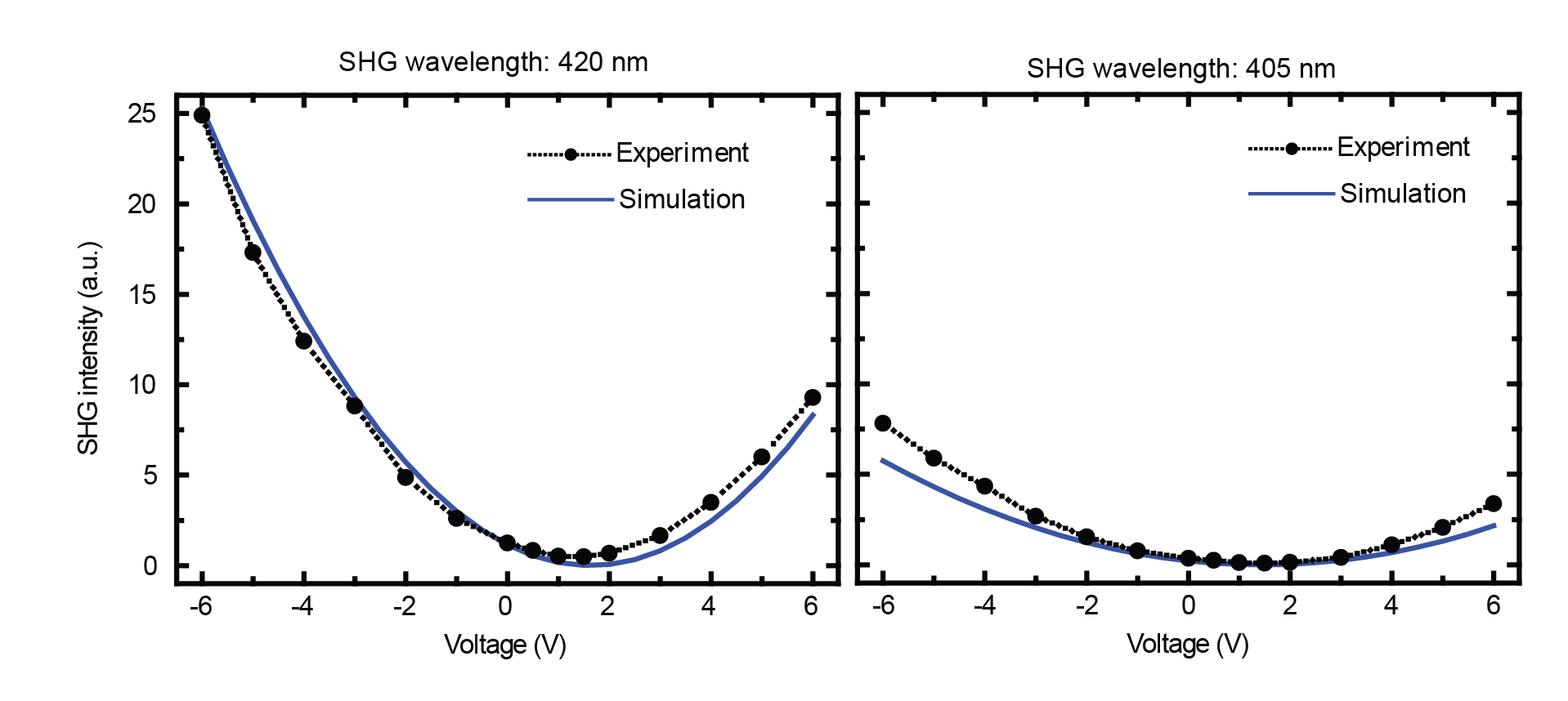
**

**Figure S2**. Nonlinear optical properties of ITO/PFO/Aluminium with applied voltages. For SHG wavelength at 420 nm and 405 nm, the SHG intensity as a function of the applied voltages is plotted. In the case of positive and negative voltages, ITO layer serves as anode and cathode, respectively.

To better understand the measured EFISH results from the ITO/PFO/Aluminum sandwiched device, the nonlinear polarization distribution that contributes to SHG in the far-field was calculated based on the linear optical responses at the fundamental and second harmonic waveelengths^1,2^. The distribution of fundamental electric field is simulated for H-polarized incident plane wave at 45^o^ incident angle. The inversion symmetry of the optical setup is broken by the oblique incidence of FW, resulting in SHG at the ITO/PFO and PFO/Aluminum interfaces. In this configuration, the non-zero second order susceptibilities of the interfaces are$\chi_{zzz}$, $\chi_{zxx}=\chi_{zyy}$and$\chi_{xxz}=\chi_{yyz}=\chi_{yzy}=\chi_{xzx}$, where the value of$\chi_{zzz}$ is usually two orders larger than others^3^. For simplicity, only the $E_{z}$ component needs to be considered in the calculation of the nonlinear polarization, namely,$P_{SHG}=\chi^{(2)}\cdot E_{z}E_{z}$, with $\chi^{(2)}\sim\chi_{zzz}$. Similarly, the EFISH process involves only $E_{z}$and $E_{DC}$ because a zero dot product between the z-oriented $E_{DC}$with the x, y components of the FW electric field. Therefore, with the third-order nonlinear coefficient $\chi^{(3)}$ of isotropic PFO thin film, we obtain $P_{EFISH}=\chi^{(3)}\cdot E_{z}E_{z}E_{DC}$. The contribution to the far field SHG signal from nonlinear polarizations of SHG and EFISH at each local point $P(r)$ can be calculated by

$\vec{E}_{total}(r')=\int\overleftrightarrow{G}(r',r)\cdot(\vec{P}_{EFISH}(r)+\vec{P}_{SHG}(r))d^{3}r$. (2)

where $\overleftrightarrow{G}(r',r)$ is the green function. In Fig. S2 we plotted the simulated total SHG intensity $|\vec{E}_{total}(r')|^{2}$ with applied DC voltage from -6 V to 6 V for fundamental wavelength at 840 nm and 810 nm, respectively. The effective$\chi^{(2)}$ and $\chi^{(3)}$ coefficients used in the simulation are retrieved from the experimental results (black circles with dot line in Fig. S2). The simulated results agree well with the experimental ones, which confirms that in this system, the EFISHG process depends on both $\chi^{(2)}$ and $\chi^{(3)}$ coefficients.


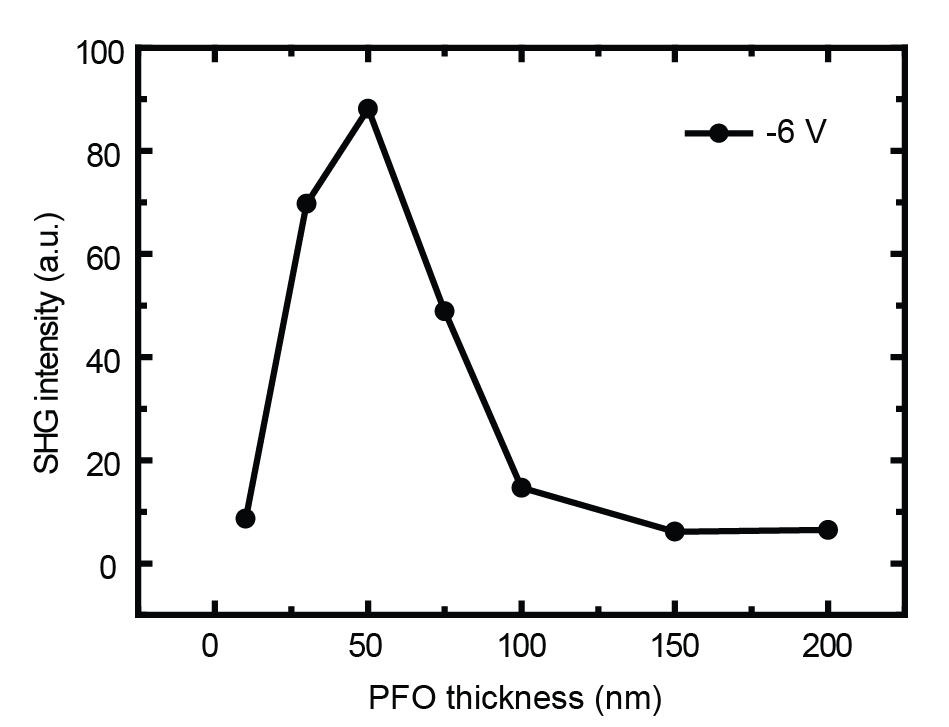


**Figure S3.** Calculated SHG intensity as a function of the thickness of PFO. The wavelength of the pumping laser is 840 nm, and the external applied DC voltage is -6 V.

The relationship between the EFISH efficiency and the thickness of PFO is also studied by using the above nonlinear optical calculation method. In Fig. S3 we plot the intensity of SHG as a function of the thickness of PFO with a fundamental wave of 840 nm and an external DC voltage of 6 V. In this case, the optimized thickness of PFO for the EFISH device is 50 nm, with the EFISH efficiency 6 times larger than that of the device with 100 nm thickness PFO. However, because of the rough surface of the Aluminum layer and ITO layer, short circuit effect has high chance to happen if the thickness of the PFO layer is less than 80 nm. That is why we choose 100 nm thick PFO in this work.

**The field distribution of fundamental wave**

**
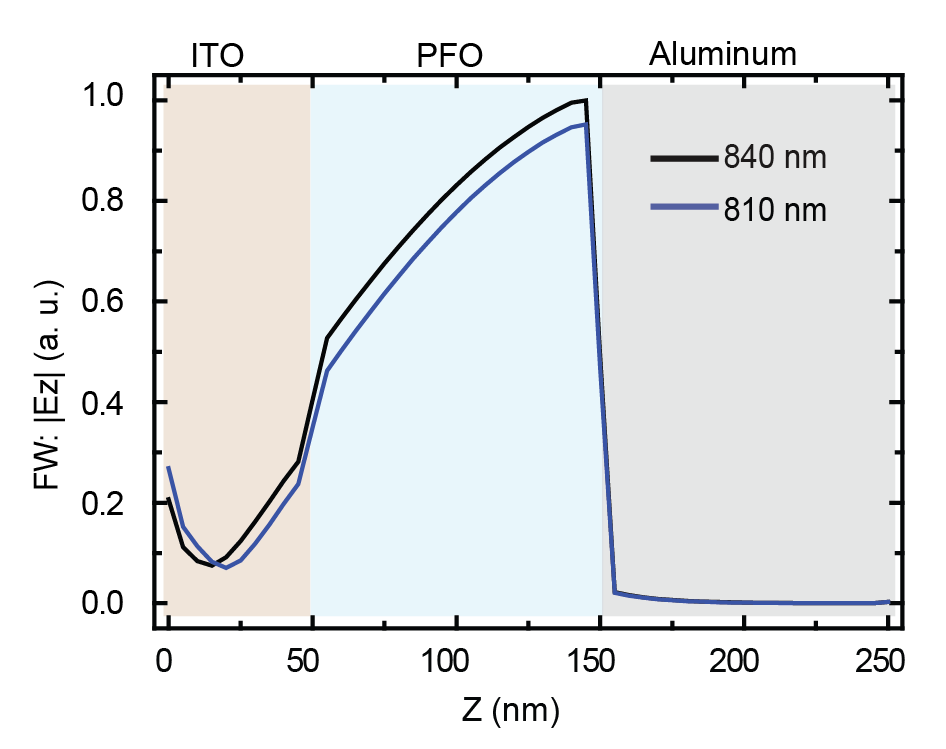
**

**Figure S4.** Calculated amplitude distributions of the z component TM-polarized fundamental wave |E_z_| at wavelength of 840 nm and 810 nm with an incident angle of 45 deg. The position of ITO, PFO and Aluminium layers are 0-50 nm, 50 nm - 150 nm and 150 nm - 250 nm, respectively.

The amplitude distribution of fundamental electric field z component $|\overrightarrow{E}_{z}|$is simulated in the case of H-polarized incident plane wave at 45 degree incident angle. As shown in Fig. S4, for both 840 nm and 810 nm FW, the electric field is mainly localized in the 100 nm PFO layer, which confirms that the giant EFISH effect is originated from the PFO thin film.

**The Refractive Index of PFO**

**
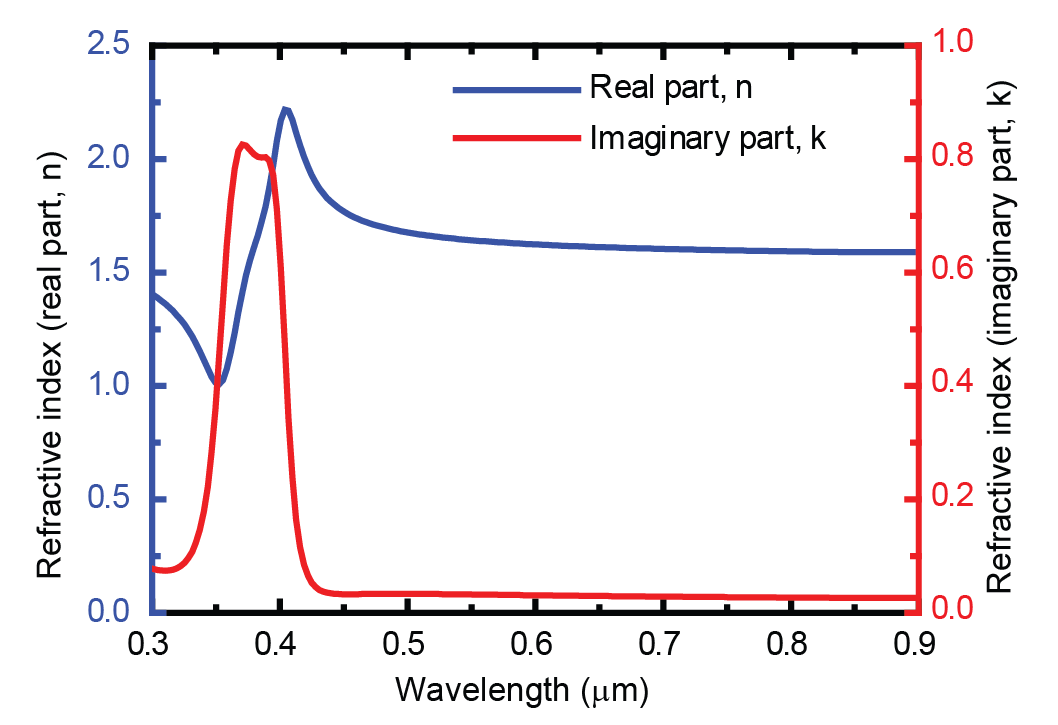
**

**Figure S5.** Refractive index of PFO measured by using spectroscopic Ellipsometer. The blue line and red line are the real part (n) and imaginary part (k) of the refractive index, respectively. The green dash line at the wavelength of 420 nm corresponds to the peak positon of the measured EFISH.

In Fig. S5, we plot the dispersion relation of PFO measured by spectroscopic Ellipsometer. The shape changes in both real part and imaginary part of the refractive index in the UV wavelength range is originated from the absorption property of PFO. The green dash line located at a wavelength of 420 nm correspond to the peak position of the measured SHG spectra shown in Fig. 4b.

**Reference**

1. O’Brien K, Suchowski H, Rho J, Salandrino A, Kante B *et al*. Predicting nonlinear properties of metamaterials from the linear response. *Nat Mater* 2015; **14**: 379-383.
2. Li GX, Chen SM, Pholchai N, Reineke B, Wong PWH *et al*. [Continuous control of the nonlinearity phase for harmonic generations](http://www.nature.com/nmat/journal/v14/n6/abs/nmat4267.html). *Nat Mater* 2015; **14**: 607-612.
3. Krause D, Teplin CW, Rogers CT. Optical surface second harmonic measurements of isotropic thin-film metals: Gold, silver, copper, aluminium and tantalum. *J. Appl. Phys.* 2004; **96**: 3626.
